# Management of metacarpal shaft fractures: a survey of current UK practice

**DOI:** 10.1308/rcsann.2024.0070

**Published:** 2024-10-08

**Authors:** R Taha, TR Davis, AA Montgomery, A Karantana

**Affiliations:** ^1^ University of Nottingham, UK; ^2^ Nottingham University Hospitals NHS Trust, UK

**Keywords:** Metacarpal bones, Hand fractures, Cross-sectional survey

## Abstract

**Introduction:**

Metacarpal shaft fractures (MSF) are common injuries that predominantly affect young, economically active people. However, there is limited evidence to guide their management. The aims of this study were to: evaluate the management of extra-articular MSF of the fingers; assess equipoise for surgical and nonsurgical treatments; and explore factors influencing clinician decision making to inform the design of a randomised controlled trial (RCT) comparing surgical and nonsurgical treatments.

**Methods:**

A cross-sectional, web-based survey was distributed to UK hand surgeons using membership directories of different professional networks. Practice setting, clinical experience, management strategies, willingness to participate in a RCT and factors affecting suitability for randomisation were recorded.

**Results:**

There were 108 responses eligible for analysis. Distribution of clinical experience ranged from <5 to >20 years. A variety of treatments were used for transverse, long oblique/spiral and comminuted MSF. Rotational deformity (90%), step-off deformity (5%) and angulation (5%) were the most important indications for surgical fixation. Acceptable limits of fracture angulation and shortening varied among surgeons. Over 85% expressed interest in participating in a RCT and most showed equipoise and were willing to offer operative or nonoperative treatment as part of a research study.

**Conclusions:**

This survey demonstrates that UK hand surgeons have varying views on treatments, acceptable parameters of deformity and indications for surgical fixation of displaced MSF. There is equipoise for surgical and nonsurgical treatments, variability in factors influencing clinical decision making and support for RCTs to investigate best practice.

## Introduction

Hand injuries are common and hand fractures represent 20% of all fractures presenting to emergency departments.^1,2^ Although they are rarely life-threatening, hand injuries are associated with significant costs, ranging from £100 million per year in the UK^3^ to €2 billion in Germany,^4^ and more than $18 billion is spent treating upper extremity injuries in the United States.^5^ The metacarpals are among the most commonly injured bones in the hand, with a lifetime incidence of 2.5%.^6,7,8^ Although metacarpal neck fractures (so called ‘boxer's fracture’) make up the majority, fractures of the shaft account for up to 44% of metacarpal fractures.^9^

Metacarpal shaft fractures (MSF) predominantly affect young adult male patients, with the ring and little finger commonly affected.^2,6,8^ They usually occur because of a direct impact to the hand, axial loading or a twisting injury.^6,7^ The aim of treatment is to restore hand function by achieving fracture union in acceptable alignment, either with operative fixation or nonoperatively, with or without manipulation. A recent systematic review confirmed the lack of high-quality evidence to guide treatment.^10^ Moreover, included studies lacked consistency and had a high or critical risk of bias.^10^ A variety of surgical and nonsurgical treatments are used, with varying modes and periods of immobilisation and a plethora of surgical fixation techniques, including open reduction and internal fixation (ORIF), lag screw fixation, cerclage wiring, intramedullary fixation and external fixation.^11–18^

Therefore, the optimal treatment for MSF is not known and treatment varies among surgeons.^19,20^ We conducted a survey of UK hand surgeons to investigate current practice and to identify factors influencing clinician decision making in the management of MSF. The aim of this study was to explore management of closed, displaced extra-articular MSF of the fingers, assess equipoise for treatments and to explore willingness to participate in a randomised controlled trial (RCT) comparing surgical with nonsurgical treatments.

## Methods

**Figure 1:**
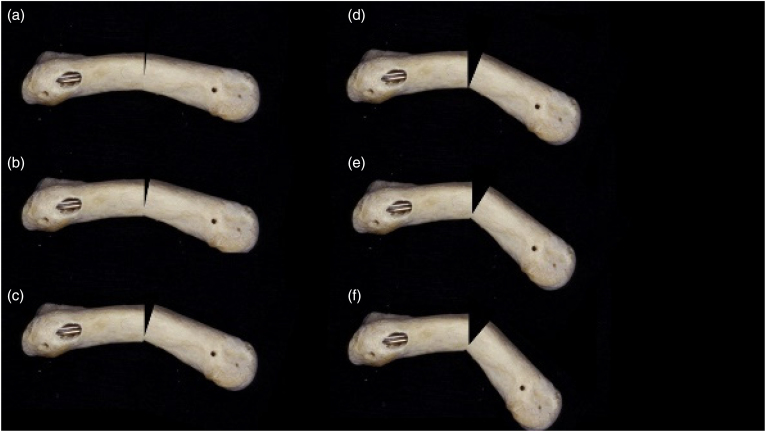
Example question from online survey. In your opinion, which of these lie in acceptable angulation and do not need to be reduced into better alignment (please select only ONE option): (a) a only; (b) a and b; (c) a–c; (d) a–d; (e) all of the above are acceptable; (f) none of the above are acceptable; (g) other, please specify.

A cross-sectional survey of UK hand surgeons was conducted using online survey software (SurveyMonkey Inc., San Mateo, CA, USA). The survey was developed by a team of clinical researchers and methodologists using a systematic four-stage approach of item generation, item reduction, formatting and pretesting. Questions were developed to explore the management of closed extra-articular MSF of the fingers. Pictorial and radiographic representation of degrees of angulation, shortening and deformity were used to explore treatment decision making (Figure 1 and Supplementary online material). The survey was piloted locally by two practising National Health Service (NHS) consultant hand surgeons who provided qualitative feedback on clarity, face validity, ease of use and acceptability to clinicians.

The survey consisted of 44 questions in 4 sections: (1) demographic details including practice setting, type of clinician (consultant, trainee surgeon or therapist) and duration of practice in years; (2) preferred treatments for common patterns of MSF, surgical and nonsurgical treatments clinicians would be willing to offer as part of a research study, suitability for randomisation, and patient and fracture factors affecting suitability for inclusion in a RCT; (3) acceptable limits of displacement and criteria for surgical intervention; and (4) feedback on the survey, such as time required for completion, acceptability to responders and a free text box inviting further comments (Supplementary online material).

**Figure 2:**
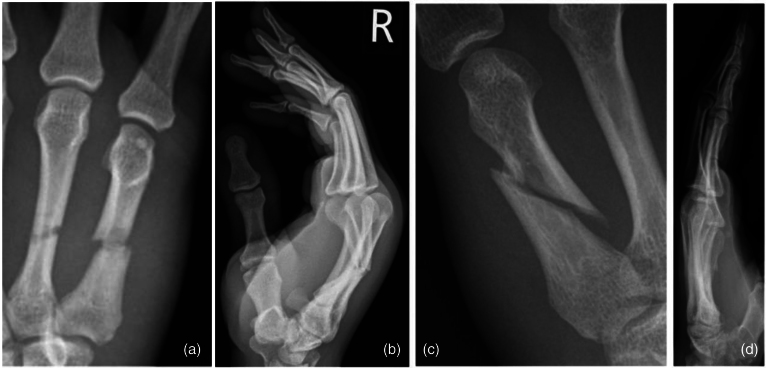
Sample of radiographs included in the survey to assess preferred treatments: (a) anteroposterior view transverse metacarpal shaft fractures (MSF) ring finger, (b) lateral view transverse MSF ring finger, (c) anteroposterior view long oblique MSF little finger and (d) lateral view long oblique MSF little finger.

Respondents were provided with radiographs of three common fracture patterns and a list of treatments from which to select their preferred treatment option for an acute displaced closed extra-articular fracture of the metacarpal shaft affecting the ring and/or little finger (examples of radiographs are provided in Figure 2, treatment options are listed in the Supplementary online material).

The survey was distributed via email, containing a hyperlink to the online form, to all members of the British Society for Surgery of the Hand (BSSH) and the Reconstructive Surgical Trials Network (RSTN). The RSTN is the national UK clinical trials network for plastic and hand surgery and consists of voluntary membership of individuals with an interest in hand surgery research.^21^ The survey was included in monthly e-newsletters and reminder emails were sent to nonresponders 6 and 12 weeks after initial invitation. Although the target group for the survey was consultant hand surgeons practising in the UK, it was not possible to identify this specific group on BSSH and RSTN email lists.

The Internet Protocol address and email of respondents were recorded to identify duplicate entries, and cookies were used to prevent multiple entries from the same individual, on the same device, in the same browser. Participation was voluntary and all answers were anonymised.

### Statistical analysis

Quantitative data were summarised using appropriate descriptive statistics using STATA (SE 16.1, StataCorp LLC, College Station, TX, USA). All responses were analysed, including incomplete surveys. Free text responses were analysed and coded into common themes and compared with quantitative data by the primary author (RT). Respondents’ hospital of practice was used to plot their geographical distribution using Google Maps™ mapping service (Map data©2021 GeoBasis-DE/BKG (©2009), Google, Inst. Geogr. National).

## Results

### Demographics

**Figure 3:**
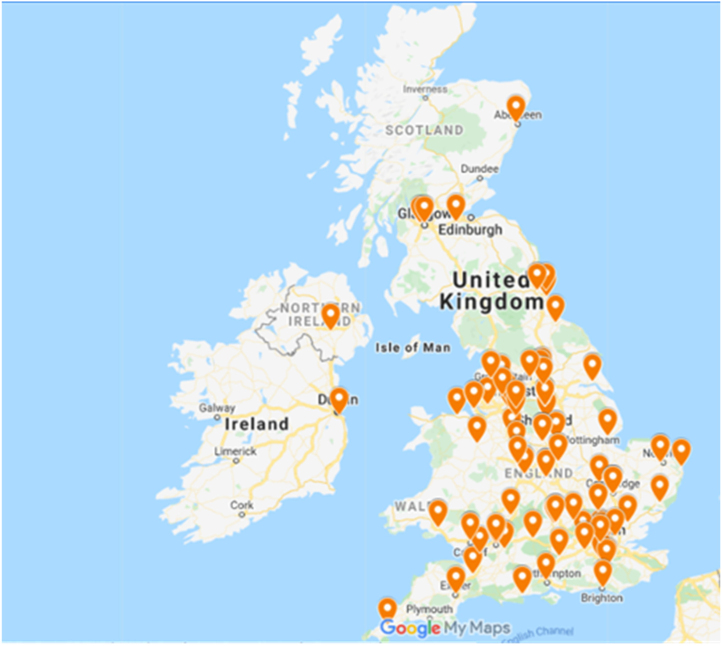
Geographical distribution of survey respondents

Of the 189 responses received, 108 were from consultant hand surgeon members of the BSSH, of whom 86 (80%) completed the entire survey. We excluded 47 responses that completed demographic details only, 16 international and 18 responses by trainee surgeons and therapists (13 and 5, respectively). The geographical distribution and years of clinical experience of respondents is displayed in Table 1 and Figure 3.

**Table 1:** Clinical experience of survey respondents

Experience as a hand surgeon (years)	Respondents (*N*=108) *n* (%)	Cumulative *n* (%)
<5	20 (19)	20 (19)
5–10	24 (22)	44 (41)
10–15	26 (24)	70 (65)
15–20	19 (18)	89 (82)
>20	19 (18)	108 (100)

### Preferred treatments

Numerous different treatments were reported for the management of MSF and treatment varied according to fracture pattern (Table 2). When broadly classified into nonoperative, percutaneous fixation and internal fixation, internal fixation was the preferred choice of treatment for transverse MSF (45%). Nonoperative treatments were preferred for long oblique/spiral (58%) and comminuted MSF (53%). Open reduction and dorsal plate fixation was the most commonly used method of surgical fixation overall; however, lag screw fixation was favoured for long oblique/spiral MSF. Early mobilisation without a cast or splint was the most frequently used nonoperative intervention. Numbers of MSF varied, with most seeing an average of four to six per month of the three fracture patterns included in the survey (Table 3).

**Table 2:** Preferred treatment for acute closed extra-articular metacarpal shaft fractures affecting the finger digits (respondents *N*=108)

Treatment	Fracture pattern		
	Transverse *n* (%)	Long oblique/spiral *n* (%)	Comminuted *n* (%)
Open operative techniques			
Open reduction and dorsal plate fixation	46 (44)	6 (6)	27 (31)
Open reduction and lag screw fixation	0	26 (28)	9 (10)
Open reduction and longitudinal (intramedullary) K-wire(s)	3 (3)	0	0
Other ORIF	4 (4)	2 (2)	0
Total ORIF	53 (49)	34 (35)	36 (41)
Percutaneous operative techniques			
Closed reduction + percutaneous longitudinal (intramedullary) K-wire(s)	8 (8)	0	0
Closed reduction + percutaneous transverse K-wires	1 (1)	1 (1)	1 (1)
Closed reduction + percutaneous transverse K-wires acrossfracture fragments and into adjacent intact metacarpal	1 (1)	2 (2)	1 (1)
External fixation	0	0	2 (2)
Other percutaneous	0	0	0
Total percutaneous	10 (9)	3 (3)	4 (5)
Nonoperative techniques			
Early mobilisation, if pain allows	7 (7)	29 (31)	20 (23)
Immobilisation without fracture reduction using a cast/splint whichleaves the fingers free	1 (1)	17 (18)	6 (7)
Closed reduction and immobilisation in a short hand cast, which allowsfree mobilisation of the wrist, MCP, PIP and DIP joints	15 (14)	4 (4)	5 (6)
Closed reduction and immobilisation in a cast/splint which leaves the fingers free	14 (13)	2 (2)	9 (10)
Immobilisation without fracture reduction using a cast/splint whichincorporates the fingers and prevents their use	0	5 (5)	3 (3)
Closed reduction and immobilisation in a cast/splint which incorporatesthe fingers and prevents their use	5 (5)	0	3 (3)
Other nonoperative	0	0	0
Total nonoperative	42 (39)	57 (58)	46 (53)
Other^a^	3 (3)	4 (4)	1 (1)

DIP = distal interphalangeal; K-wire = Kirschner wire; MCP = metacarpophalangeal; ORIF = open reduction internal fixation; PIP = proximal interphalangeal.

Percentages may not sum to 100 because of rounding. Other open surgical techniques include intramedullary compression screw fixation and intraosseous figure-of-eight wiring for transverse fractures, ulnar cortex plate fixation and combined lag screw and plate fixation for long oblique/spiral fractures.

^a^Other responses were not included in denominator, respondents indicated that treatment varied according to patient characteristics and/or multiplicity of fractures.

**Table 3:** Estimated number of metacarpal shaft fractures per month (respondents *N*=108)

	Fracture pattern			Total *n* (%)
	Transverse *n* (%)	Long oblique/spiral *n* (%)	Comminuted *n* (%)	
Estimated number seen at unit per month				
<1	6 (6)	6 (6)	15 (17)	27 (9)
1	9 (8)	13 (13)	23 (26)	45 (15)
2–3	40 (37)	34 (35)	29 (33)	103 (35)
4–6	22 (20)	22 (23)	17 (20)	61 (21)
7–10	22 (20)	15 (15)	3 (3)	40 (14)
11–15	2 (2)	5 (5)	0	7 (2)
>15	7 (7)	3 (3)	0	10 (3)

### Indications for surgical intervention

**Figure 4:**
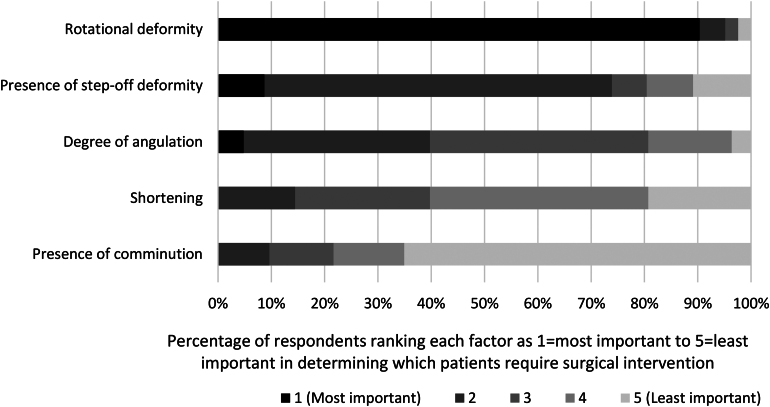
Criterion for determining which patients require surgical intervention. Presence of comminution is defined as more than two fracture fragments.

The most important criterion for surgical intervention was rotational deformity (selected by 90% of respondents), followed by presence of step-off deformity (5%) and angulation (5%) (Figure 4). However, acceptable angulation varied with over half accepting up to 30° (Table 4). There was minimal change in acceptable angulation following an attempted closed reduction (Table 4). The main reasons given for reducing a displaced MSF were loss of function (80%), cosmesis (8%) and other reasons (12%), such as both cosmesis and loss of function, rotational deformity, angulation, presence of three or more MSF and patient characteristics.

**Table 4:** Deformity reported as acceptable for acute extra-articular fractures of the metacarpal shaft affecting the ring and/or little finger (respondents *N*=108)

Deformity	Acceptable *n* (%)	Cumulative *n* (%)
Acceptable angulation (°)^a^		
>40	6 (11)	6 (8)
40	19 (24)	25 (32)
30	28 (35)	53 (67)
20	23 (29)	76 (96)
10	3 (4)	79 (100)
None	0	
Other^a^	3	
Acceptable angulation following attempted closed reduction (°)^b^		
>40	5 (6)	5 (6)
40	16 (20)	21 (26)
30	28 (34)	49 (60)
20	30 (37)	79 (96)
10	3 (4)	82 (100)
None	0	
Other^a^	1	
Shortening (mm)^c^		
>5	33 (47)	33 (47)
4	14 (20)	47 (67)
3	16 (23)	63 (90)
2	4 (6)	67 (96)
None	3 (4)	
Other^a^	13	

^a^Question: In your opinion, which of these lie in acceptable angulation and do not need to be reduced into better alignment (please select only ONE option).

^b^Question: Having attempted a closed reduction of the fracture, which of the following would you accept? (Please select only ONE option).

^c^Question^:^ If you use shortening as a criterion for surgical management, how much shortening (mm) is acceptable? (Please select only ONE option). Other responses were not included in analyses.

Of surgeons who use shortening as a criterion for surgical fixation, 47% accept up to 5mm, 6% are more restrictive accepting up to 2mm and 4% consider any amount of shortening to be unacceptable (Table 4). In addition, 65% of surgeons reported that the presence of shortening with step-off deformity would change their view on management of transverse MSF.

### Participation in a RCT comparing treatment interventions for MSF

Most surgeons were willing to participate in a RCT comparing surgical and nonsurgical treatments and would be comfortable offering either form of treatment within a research study, with minimal variation across fracture patterns (Table 5).

**Table 5:** Participation in a randomised controlled trial (RCT) comparing treatment interventions for metacarpal shaft fractures (respondents *N*=108)

	Fracture pattern		
	Transverse *n* (%)	Long oblique/spiral *n* (%)	Comminuted *n* (%)
Participation in a RCT comparing operative and nonoperative treatment			
Willing to participate in RCT	95 (88)	85 (87)	75 (86)
Not willing to participate in RCT	13 (12)	13 (13)	12 (14)
Operative and nonoperative treatment			
Comfortable treating such fractures operatively OR nonoperatively	95 (88)	83 (85)	77 (89)
Operative treatment			
Comfortable treating such fractures operatively	108 (100)	90 (92)	83 (95)
Not comfortable treating such fractures operatively, but have a colleague who is	0	2 (2)	4 (5)
Not comfortable treating such fractures operatively, and do not have a colleague who is	0	6 (6)	0
Nonoperative treatment			
Comfortable treating such fractures nonoperatively	95 (88)	89 (91)	79 (91)
Not comfortable treating such fractures nonoperatively, but have a colleague who is	5 (5)	5 (5)\	1 (1)
Not comfortable treating such fractures nonoperatively, and do not have a colleague who is	8 (7)	4 (4)	7 (8)

**Figure 5:**
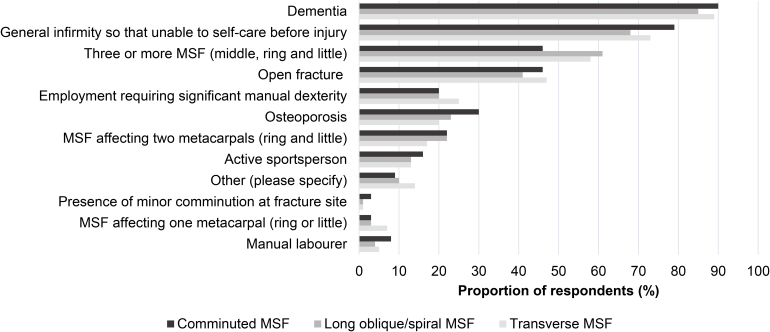
Patient characteristics which respondents would not be willing to include in a randomised study comparing surgical vs nonsurgical treatment. MSF = metacarpal shaft fracture. Other reasons cited were degree of angulation, presence of rotational deformity, inability to understand study procedures, current smoking history and comorbidities such as epilepsy, significant psychiatric illness or polytrauma.

We asked respondents to select which factors would, in their view, make a patient unsuitable for inclusion in a randomised study comparing operative and nonoperative treatment. Dementia, general infirmity so that unable to care for one's self preinjury, and the presence of three or more MSF were the most frequently selected factors across all fracture patterns (Figure 5).

## Discussion

This national survey is one of few studies exploring surgical decision making and treatment preferences for MSF.^19,20^ It demonstrates widespread variability in the treatment of MSF of the finger digits among UK hand surgeons with no consensus on treatment modality, method of immobilisation or surgical fixation technique. There was also no agreement on acceptable deformity in MSF, with variation in angulation, shortening and indications for surgical intervention. The findings confirm there is uncertainty within the clinical community, in line with Freedman's evaluation of clinical equipoise, and provides support for a randomised trial comparing surgical and nonsurgical treatments for the three common MSF patterns (transverse, long oblique/spiral and multifragmentary).^22,23^

Previous surveys conducted in metacarpal fractures evaluated a heterogeneous group of metaphyseal base, neck and shaft fractures, which impedes translation of their findings to MSF as neck fractures are more common and tolerate greater deformity.^6,12,19,20,24^ One was conducted in Canadian plastic surgeons, the second included UK upper limb surgeons, and the third, a postal survey of Welsh surgeons, focused on boxer's fractures only.^19,20,24^ They focused on single metacarpal fractures, or the little finger metacarpal only, thus limiting the generalisability of their findings.^19,20^

Another informative finding concerns parameters of deformity in MSF because ‘acceptable’ deformity varies greatly in the literature depending on the digit affected, ranging from 5°–20° in the index and middle finger to 40°–60° at the ring and little finger.^11,25^ Similar differences also exist for shortening, with some accepting 2mm and others up to 5mm before proceeding to surgical intervention.^25–28^ Although there was no consensus in our survey on acceptable deformity, we identified watershed levels (selected by over half of respondents) at 30° of angulation and between 4 and 5mm of shortening (for the ring and little finger) that may be suitable cut-offs for use in future studies. This contrasts with findings by Sahu *et al* that 39% of surgeons tolerate 30° of angulation before intervention, whereas (cumulatively) 67% accepted such angulation in the current study. Surgeons tolerated far greater shortening in our survey than previously reported, with 47% accepting up to 5mm in contrast to 21% reported by Retrouvey and colleagues.^19^ Rotational deformity, although unusual with these fractures, at presentation was uniformly considered the most important indication for surgical intervention, despite excellent outcomes reported by Khan and Giddins in their series following nonoperative treatment of spiral metacarpal fractures in the presence of initial malrotation.^27^ However, the assessment of rotational deformity in MSF is difficult because of the high incidence of pseudorotation caused by rotation of the metacarpal secondary to intermetacarpal swelling in the presence of fracture.^29^ Other indications reported in the literature, such as multiplicity of fractures or segmental bone loss, were not supported by the current survey, highlighting differences in expert opinion.^25,30^ In addition, the description and reporting of parameters of deformity in MSF need to be clearly and consistently reported to facilitate pooling of data and allow for meaningful comparison of outcomes in future studies.

### Study limitations

Our findings are limited by the restriction to UK consultants, risk of recall bias of respondents and, as with any survey, the self-selection of respondents. Because we utilised membership directories of different professional societies, it was not possible to establish an accurate denominator to calculate a response rate. Although the BSSH is the largest professional body of hand surgeons in the UK, membership includes surgeons in training, therapists, retired surgeons and international members. In addition, some members may not treat hand fractures in adults due to having a purely elective, private or predominantly paediatric practice.

However, this is balanced by the broad spectrum of years of clinical experience, variety of practice settings (district hospitals, large academic institutions and specialist tertiary hand surgery units) and wide geographical distribution of respondents. Furthermore, this national survey addresses the limitations of previous surveys by investigating factors influencing preferred treatments for specific fracture patterns, allowing assessment of the subtleties in management and exploration of the impact of fracture characteristics on the treatment of MSF. It also identifies a variety of patient and fracture characteristics that may affect suitability for randomisation, helping to inform eligibility criteria for future studies, while recording the number of MSF per unit will help inform selection of sites and facilitate collaborative, multi-centre research.

## Conclusion

This survey highlights considerable heterogeneity in the management of MSF, and variation in treatment strategies and factors determining treatment by UK hand surgeons. It confirms that there is equipoise for surgical and nonsurgical treatments, support for randomised comparative trials to inform best practice and identifies areas for further research to optimise their design.

## References

[1] van Onselen EB, Karim RB, Hage JJ, Ritt MJ. Prevalence and distribution of hand fractures. *J Hand Surg Br* 2003; **28**: 491–495. 12954264 10.1016/s0266-7681(03)00103-7

[2] Johansen A, Evans RJ, Stone MD *et al.* Fracture incidence in England and Wales: a study based on the population of Cardiff. *Injury* 1997; **28**: 655–660. 9624346 10.1016/s0020-1383(97)00144-7

[3] Dias JJ, Garcia-Elias M. Hand injury costs. *Injury* 2006; **37**: 1071–1077. 17045996 10.1016/j.injury.2006.07.023

[4] Liener UC, Rapp U, Lampl L *et al.* Inzidenz schwerer Verletzungen. Ergebnisse einer populationsbezogenen Untersuchung [Incidence of severe injuries. Results of a population-based analysis]. *Unfallchirurg* 2004; **107**: 483–490. German. 15170503 10.1007/s00113-004-0771-5

[5] Kelsey J, Praemer A, Nelson L *et al.* *Upper Extremity Disorders: Frequency, Impact and Cost*. New York: Churchill Livingstone; 1998. 86 p.

[6] Stanton JS, Dias JJ, Burke FD. Fractures of the tubular bones of the hand. *J Hand Surg Eur* 2007; **32**: 626–636. 10.1016/J.JHSE.2007.06.017 17993422

[7] Feehan LM, Sheps SB. Incidence and demographics of hand fractures in British Columbia, Canada: a population-based study. *J Hand Surg Am* 2006; **31**: 1068–1074. 16945705 10.1016/j.jhsa.2006.06.006

[8] de Jonge JJ, Kingma J, van der Lei B, Klasen HJ. Fractures of the metacarpals. A retrospective analysis of incidence and aetiology and a review of the English-language literature. *Injury* 1994; **25**: 365–369. 8045639 10.1016/0020-1383(94)90127-9

[9] Chung KC, Spilson SV. The frequency and epidemiology of hand and forearm fractures in the United States. *J Hand Surg Am* 2001; **26**: 908–915. 11561245 10.1053/jhsu.2001.26322

[10] Taha RHM, Grindlay D, Deshmukh S *et al.* A systematic review of treatment interventions for metacarpal shaft fractures in adults. *Hand (N Y)* 2020; **17**: 869–878. 33252278 10.1177/1558944720974363 PMC9465778

[11] Kozin SH, Thoder JJ, Lieberman G. Operative treatment of metacarpal and phalangeal shaft fractures. *J Am Acad Orthop Surg* 2000; **8**: 111–121. 10799096 10.5435/00124635-200003000-00005

[12] Giddins GE. The non-operative management of hand fractures. *J Hand Surg Eur* 2015; **40**: 33–41. 10.1177/1753193414548170 25217094

[13] Al-Qattan MM. Outcome of conservative management of spiral/long oblique fractures of the metacarpal shaft of the fingers using a palmar wrist splint and immediate mobilisation of the fingers. *J Hand Surg Eur* 2008; **33**: 723–727. 10.1177/1753193408093559 18662959

[14] Debnath UK, Nassab RS, Oni JA, Davis TR. A prospective study of the treatment of fractures of the little finger metacarpal shaft with a short hand cast. *J Hand Surg Br* 2004; **29**: 214–217. 15142689 10.1016/j.jhsb.2004.02.020

[15] Tavassoli J, Ruland RT, Hogan CJ, Cannon DL. Three cast techniques for the treatment of extra-articular metacarpal fractures. Comparison of short-term outcomes and final fracture alignments. *J Bone Joint Surg Am* 2005; **87**: 2196–2201. 16203883 10.2106/JBJS.D.03038

[16] Faraj AA, Davis TR. Percutaneous intramedullary fixation of metacarpal shaft fractures. *J Hand Surg Br* 1999; **24**: 76–79. 10190611 10.1016/s0266-7681(99)90039-6

[17] Al-Qattan MM. Metacarpal shaft fractures of the fingers: treatment with interosseous loop wire fixation and immediate postoperative finger mobilisation in a wrist splint. *J Hand Surg Br* 2006; **31**: 377–382. 16716472 10.1016/j.jhsb.2006.03.166

[18] Corkum JP, Davison PG, Lalonde DH. Systematic review of the best evidence in intramedullary fixation for metacarpal fractures. *Hand (NY)* 2013; **8**: 253–260. 10.1007/s11552-013-9531-8 PMC3745242 24426931

[19] Retrouvey H, Morzycki A, Wang AMQ, *et al.* Are we over treating hand fractures? Current practice of single metacarpal fractures. *Plast Surg (Oakv)* 2018; **26**: 148–153. 30148125 10.1177/2292550318767926 PMC6100138

[20] Sahu A, Gujral SS, Batra S *et al.* The current practice of the management of little finger metacarpal fractures–a review of the literature and results of a survey conducted among upper limb surgeons in the United Kingdom. *Hand Surg* 2012; **17**: 55–63. 22351534 10.1142/S0218810412500098

[21] Reconstructive Surgical Trials Network (RTSN). http://reconstructivesurgerytrials.net/ (cited June 2021).

[22] Freedman B. Equipoise and the ethics of clinical research. *N Engl J Med* 1987; **317**: 141–145. 3600702 10.1056/NEJM198707163170304

[23] Karlawish JH, Lantos J. Community equipoise and the architecture of clinical research. *Camb Q Healthc Ethics* 1997; **6**: 385–396. 9292216 10.1017/s0963180100008136

[24] Jones R, Burdett S, Jefferies M, Guha AR. Treating the boxer’s fracture in Wales: a postal survey. *Ann R Coll Surg Engl* 2010; **92**: 236–239; quiz 239. 20223051 10.1308/003588410X12628812458176 PMC3080065

[25] Wong VW, Higgins JP. Evidence-based medicine: management of metacarpal fractures. *Plast Reconstr Surg* 2017; **140**: 140e–151e. 10.1097/PRS.0000000000003470 28654615

[26] Diaz-Garcia R, Waljee JF. Current management of metacarpal fractures. *Hand Clin* 2013; **29**(4): 507–518. 24209950 10.1016/j.hcl.2013.09.004

[27] Khan A, Giddins G. The outcome of conservative treatment of spiral metacarpal fractures and the role of the deep transverse metacarpal ligaments in stabilizing these injuries. *J Hand Surg Eur* 2015; **40**: 59–62. 10.1177/1753193414540408 24963083

[28] Strauch RJ, Rosenwasser MP, Lunt JG. Metacarpal shaft fractures: the effect of shortening on the extensor tendon mechanism. *J Hand Surg Am* 1998; **23**: 519–523. 9620194 10.1016/S0363-5023(05)80471-X

[29] Smith NC, Moncrieff NJ, Hartnell N, Ashwell J. Pseudorotation of the little finger metacarpal. *J Hand Surg Br* 2003; **28**: 395–398. 12954244 10.1016/s0266-7681(03)00144-x

[30] Omokawa S, Fujitani R, Dohi Y *et al.* Prospective outcomes of comminuted periarticular metacarpal and phalangeal fractures treated using a titanium plate system. *J Hand Surg Am* 2008; **33**: 857–863. 18656755 10.1016/j.jhsa.2008.01.040

